# *Legionella pneumophila* Risk from Air–Water Cooling Units Regarding Pipe Material and Type of Water

**DOI:** 10.3390/microorganisms11030638

**Published:** 2023-03-02

**Authors:** Enrique Gea-Izquierdo, Ángel Gil-de-Miguel, Gil Rodríguez-Caravaca

**Affiliations:** 1 Preventive Medicine and Public Health, Rey Juan Carlos University, 28922 Madrid, Spain; 2 Maria Zambrano Program, European Union, Spain; 3 CIBER of Respiratory Diseases (CIBERES), Instituto de Salud Carlos III, 28029 Madrid, Spain; 4 Department of Preventive Medicine, Hospital Universitario Fundación Alcorcón, Universidad Rey Juan Carlos, 28922 Madrid, Spain

**Keywords:** legionellosis, health regulations, pipe material, type of water

## Abstract

Legionellosis is a respiratory disease related to environmental health. There have been manifold studies of pipe materials, risk installations and legionellosis without considering the type of transferred water. The objective of this study was to determine the potential development of the causative agent *Legionella pneumophila* regarding air–water cooling units, legislative compliance, pipe material and type of water. Forty-four hotel units in Andalusia (Spain) were analysed with respect to compliance with Spanish health legislation for the prevention of legionellosis. The chi-square test was used to explain the relationship between material–water and legislative compliance, and a biplot of the first two factors was generated. Multiple correspondence analysis (MCA) was performed on the type of equipment, legislative compliance, pipe material and type of water, and graphs of cases were constructed by adding confidence ellipses by categories of the variables. Pipe material–type of water (*p* value = 0.29; *p* < 0.05) and legislative compliance were not associated (*p* value = 0.15; *p* < 0.05). Iron, stainless steel, and recycled and well water contributed the most to the biplot. MCA showed a global pattern in which lead, iron and polyethylene were well represented. Confidence ellipses around categories indicated significant differences among categories. Compliance with Spanish health legislation regarding the prevention and control of legionellosis linked to pipe material and type of water was not observed.

## 1. Introduction

Legionellosis is a generic term describing the pneumonic and nonpneumonic forms of infection with the *Legionella* species of bacteria. Legionellosis varies in severity from mild to serious and can sometimes be fatal [1]. Infections with *Legionella* bacteria include Legionnaires’ disease and Pontiac fever. Legionnaires’ disease is a serious type of pneumonia (lung infection), while Pontiac fever is a milder infection that usually improves without medical care [2]. Legionnaires’ disease is an important, relatively uncommon, yet well-known form of atypical community-acquired pneumonia (CAP) and hospital-acquired pneumonia (HAP). Several factors increase the risk of contracting Legionnaires’ disease, including age over 50 years, chronic cardiovascular disease, underlying respiratory disease, chronic renal disease, diabetes, smoking, any immunosuppressive condition, travel history, and certain types of employment [3]. Although it has a low incidence, Legionnaires´ disease is an important cause of pneumonia. Among community-acquired cases, an increasing number were reported to be linked to the occupational setting, indicating the need for better recognition of work activities that increase the risk of legionellosis [4].

The genus *Legionella* comprises a large group of bacteria that inhabit various aquatic systems, and it poses a serious threat to human health and life [5] since more than 20 species can cause legionellosis, with *Legionella pneumophila* being responsible for the majority of cases. The ability to colonize diverse ecosystems makes the eradication of these microorganisms difficult. A detailed understanding of the *Legionella* habitat may be helpful in the effective control of this pathogen [6]. Some of the *Legionella* environments include natural (lakes, groundwater, rivers, compost, soil) and anthropogenic (fountains, air humidifiers, water supply systems) environments [7].

Bioaerosols from air–water cooling units are often suspected to cause community-acquired legionellosis outbreaks [8]. Although *Legionella* infections can mostly be assigned to emissions sources, uncertainty exists about the release and distribution of bacteria into the air, the occurrence of the respirable virulent form and the level of the infective concentration. To prevent and control *Legionella* contamination of air–water cooling units, maintenance actions should focus on low-emissions cleaning procedures combined with control measurements of water and air samples [9]. Procedures allowing for rapid detection and risk assessment in cases of outbreaks are essential for adequate public health control [10]. Systematic registration of equipment facilitates the identification of the sources of outbreaks and helps to shorten their duration [11].

In Spain, the epidemiological surveillance of legionellosis is preferably based on the National Epidemiological Surveillance Network. Additionally, Royal Decree 487/2022 [12] established sanitary criteria for the prevention and control of legionellosis. This legislation clarifies that certain equipment must undergo maintenance, prevention and water control, among other procedures. In this scenario, not only the type of equipment but also other elements that may influence the appearance and development of the bacteria are important. In particular, air–water cooling units, together with legislation, the type of water transferred, and the materials of the installations, play a crucial role [13,14,15].

This study aimed to determine whether the potential development of *Legionella pneumophila* may be related to air–water cooling unit materials from hotels, and it evaluated compliance with Spanish health legislation for the prevention of legionellosis.

## 2. Materials and Methods

In Spain (Andalusia, Malaga), 44 air–water cooling units (33 cooling towers, 6 evaporative condensers and 5 adiabatic condensers) located in both inland and coastal communities were analysed, and the results obtained were compared with the Spanish regulation standards through which sanitary criteria were established for the prevention and control of legionellosis [12]. A descriptive study suggested gathering information through a self-administered survey distributed to directors and individuals in charge of buildings with facilities at risk, as well as drawing water samples for a later analysis in laboratories authorized by the Spanish Sanitation Authority, in accordance with standardized regulations [16]. The minimum sampling frequencies for air–water cooling units were quarterly (*Legionella*) and monthly (total aerobic count). The determination of *Legionella* was performed using the ISO 11731 standard [16]; total aerobes were determined with the ISO 6222 standard [17] and with values greater than 10,000 CFU/mL in the monthly analysis; the efficacy of the dose and type of biocide used were verified, and *Legionella* sampling was performed. Forty variables were selected for the sanitary control of *Legionella pneumophila* development [18]: unit availability, unit operation, unit working, cooling towers, evaporative condensers, adiabatic condensers, humidifiers/evaporative cooling units/others, origin of the water, type of water, notification to the corresponding health authority, modifications or improvements in the installation, control quality of the water, analysis in an approved laboratory, distance (air–water cooling units/people exposed), distance (air–water cooling units/air conditioning or ventilation intakes), cooling system drain, flow of circulating water, flow of dragged water, continuous biocide dosing system, chlorination, ultraviolet radiation, ozonation, bromination, copper/silver ionization, physical/chemical water controls, microbiological water controls, periodicity of physical/chemical analysis, periodicity of *Legionella* analysis, periodicity of total aerobe analysis, facility maintenance programs, maintenance registries, staff training courses, external companies, companies registered, safe access, pipe materials, temperature of the sanitary cold water system, temperature of the sanitary hot water system, chemical products approved, and safety data sheets.

Exploratory data analysis and visualization of contingency tables were performed to study the dependence between variables, specifically equipment, legislative compliance, pipe material and type of water. Special attention was given to the materials, type of water and legislative compliance. Considerable dependency between materials and legislative compliance was explained. The nature of the dependence between the row and the column variables was calculated with Pearson’s residuals (standardized residuals) and the contribution (%) of each cell to the total chi-square score. Additionally, eigenvalues to determine the number of axes were considered in subsequent analyses. Furthermore, a biplot was used to explain the materials and type of water. The types of water included recycled water (Rw), wastewater treatment plant water (Wwtp) and well water (Ww). Only water from wastewater treatment plants was public. To obtain factorial solutions, principal component analysis (PA) considered the total variance, estimating the factors that contained low proportions of unique variance and, in specific cases, error variance. As an objective means of interpretation regarding air–water cooling units, a vector model was used to capture the position degrees of pipe materials on a perceptual map. Potential bacterial development was considered a function of the material and physicochemical and microbiological water controls, in addition to the frequency of said controls and, in particular, of *Legionella* spp. and total aerobes [18]. The correspondence analysis between “pipe material” and “legislative compliance” was analysed according to the observations corresponding to the number of air–water cooling units, such as a facility at risk for bacteria [19]. Finally, the chi-square test was performed for independence, in which the null hypothesis was the independence between the mentioned variables. For the correspondence analysis, the chi-square test of independence was used to analyse the frequency table formed by these variables (statistically significant, *p* < 0.05). Afterwards, interdependence was presented through dimensional reduction and a perceptual map, the latter of which was based on the association between air–water unit compliance and the materials. The observations were ordered according to legislative compliance with the Spanish regulation standards to establish sanitation criteria for the prevention and control of legionellosis: compliance (Compliance_y) and noncompliance (Compliance_n). The analysis of the observations was performed for the following pipe materials: copper (Co), lead (Le), iron (Ir), stainless steel (Ss), polybutylene (Pb), polyethylene (Pe) and polyvinyl chloride (Pv).

Multivariate analysis based on multiple correspondence analysis (MCA) as an adaptation of correspondence analysis (CA) was performed on a data table containing the 4 mentioned categorical variables. Additionally, MCA was considered a generalization of PA because the variables analysed were categorical. MCA is used to identify groups of devices with similar profiles and associations between variable categories and to reveal the most relevant variables that contribute the most to explaining the variations in the dataset [20]. The data contained 44 rows (devices) and 4 columns (categorical variables), and all were used to perform MCA. The MCA function presented a format containing a data frame with the rows and columns indicated earlier, several dimensions in the results and a logical value. In fact, the object created in the MCA contained a great deal of information presented in different matrices and lists. To interpret and visualize the MCA, the factoextra package in R was used, as well as the FactoMineR package. In particular, an analysis of the eigenvalues/variances retained by each dimension (axis) was conducted, visualizing the percentages of inertia explained by each MCA dimension and extracting the results for devices and variables. The results included the coordinates of the variables, the quality of the representation for the variable on a factor map (cos2) and the contributions (%) of the variables to the definition of the dimensions. Additionally, a plot was constructed to identify variables that were the most correlated with the MCA principal dimensions, and the squared correlations between variables and dimensions were used as coordinates. A mosaic pair plot was produced by a matrix of scatterplots that returned each pair of variables in the data frame. In the pair plot, diagonal boxes showed the variables (legislative compliance, pipe material and type of water), and all other boxes displayed a scatterplot between each pairwise combination of these variables.

Additionally, graphs of cases were constructed by adding confidence ellipses by categories of each categorical variable. First, we computed PA specifying a factor (category) for colouring the cases by groups and adding an ellipse around each group. Mean points of groups of cases (barycentres) could be observed when groups were formed. The ellipses were adapted to given plane representations to visualize whether the categories were significantly different. Analysis was conducted in R Core Team (2020) (R: A language and environment for statistical computing. R Foundation for Statistical Computing, Vienna, Austria. URL https://www.R-project.org/ (accessed on 26 February 2023).

## 3. Results

**Figure 1:**
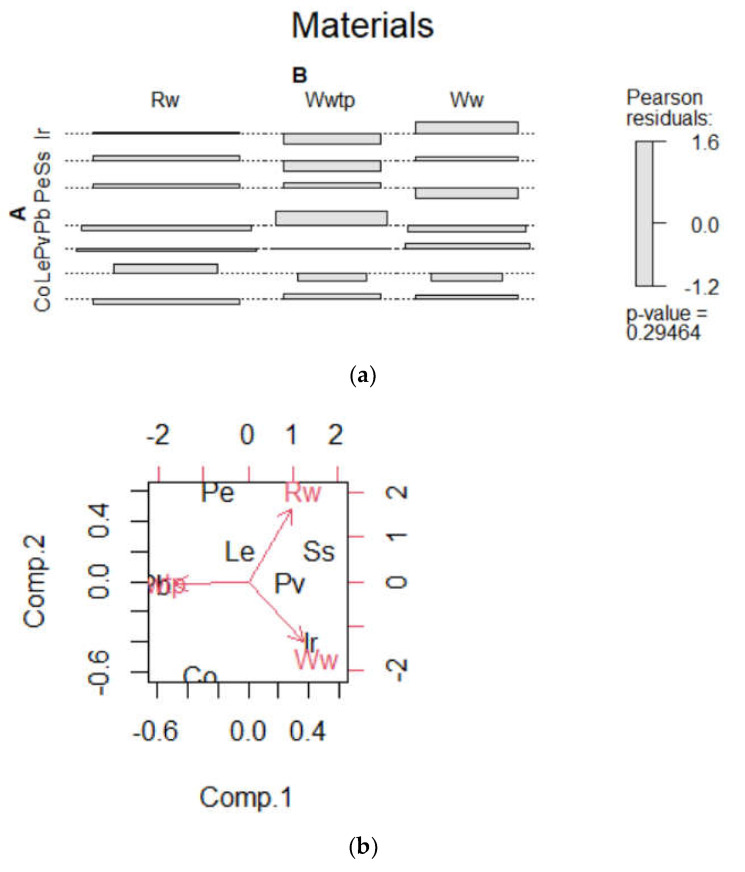
(**a**) Material–type of water, Pearson’s residuals; (**b**) Material–type of water, biplot. Recycled water (Rw), wastewater treatment plant water (Wwtp), well water (Ww). Copper (Co), lead (Le), iron (Ir), stainless steel (Ss), polybutylene (Pb), polyethylene (Pe), polyvinyl chloride (Pv).

To analyse whether there was dependence between “pipe material” and “type of water”, referred to as “legislative compliance” in air–water cooling units, contingency tables were generated. Furthermore, the chi-square test of independence showed a Pearson’s λ value = 14.09 (*p* value = 0.29), so materials and type of water were not statistically significantly associated (*p* < 0.05). Pearson’s residuals can be visualized in Figure 1a, in which the highest absolute standardized residuals contributed the most to the chi-square score (Ir > Ss > Pe > Pb). The positive values specified a positive association between pipe material and legislative compliance, and negative values implied the opposite.

In the biplot (Figure 1b), the coordinates of the pipe material and type of water are not constructed in the same space, so it is very important to focus on the direction, not on the absolute positions on the plot. The results show that materials that are on the same side of a given type of water have a high value for this category and, on the opposite side, a low value. In the contribution biplot, the points Le and Pv contribute very little to the solution because they are close to the centre and are relatively unimportant to the interpretation. The closer an arrow is (in terms of angular distance) to an axis, the greater the contribution on that axis relative to the other axis. If the arrow is halfway between the two, it contributes to the two axes to the same extent [20]. In Figure 1b, Wwtp makes a major contribution to the negative pole of the first dimension, while Rw and Ww contribute to the two axes to the same extent (positive-negative).

**Figure 2:**
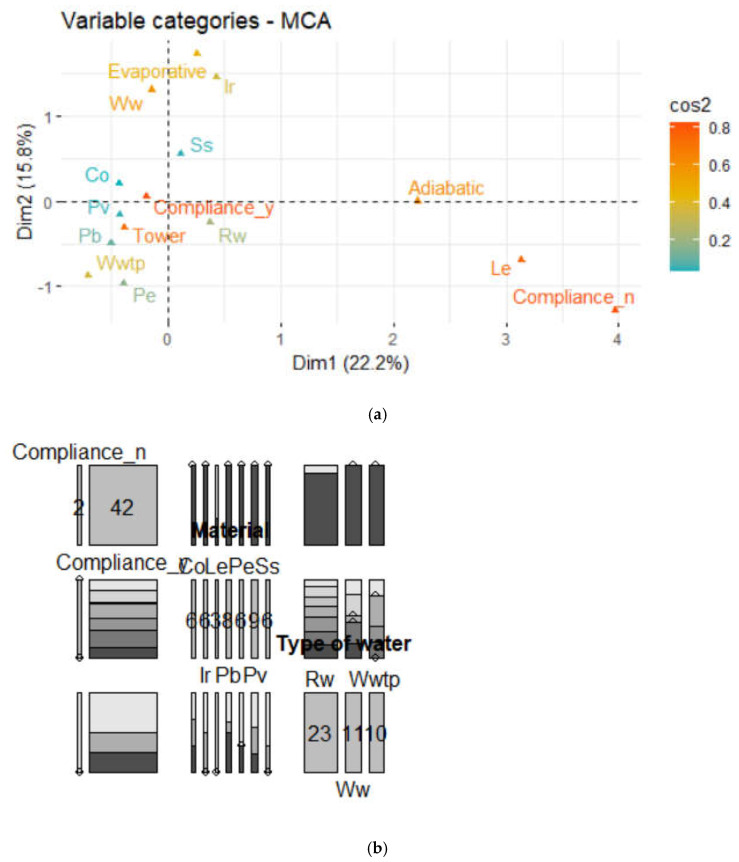
(**a**) Quality of representation of variable categories by MCA. cos2: blue (0–0.2), grey (0.2–0.4), yellow (0.4–0.6), orange (0.6–0.8); (**b**) Mosaic pair plot (legislative compliance, pipe material and type of water).

Figure 2 was used to draw the biplot of variable categories. The first plot of the MCA (Figure 2a) shows a global pattern in which variable categories are represented by triangles. The distance between any categories gives a measure of their similarity. Additionally, those with similar profiles are closed on the factor map. To extract the results for the categories, the squared cosine (cos2) was obtained. This value expresses the quality of the representation for variables on the map and measures the degree of association between categories and a specific axis. The two dimensions are enough to retain 38% of the total inertia (variation) contained in the data. Some of the points are not equally well displayed in the dimensions, but if a variable category is well represented by two dimensions, the sum of cos2 is near one. However, for some of the variable categories, more than two dimensions are required to perfectly render the data. The cos2 shows the importance of a PA for a given observation (vector of original variables). Components with a large value of cos2 contribute a relatively large portion to the total distance; therefore, these components are important for that observation. A high cos2 indicates good representation of the variable on the PA. A low cos2 indicates that the variable is not perfectly represented by the PA, so well-projected variables are interpreted with a high cos2. In Figure 2a, variable categories with low cos2 values are in blue, those with a medium cos2 are in grey, and those with a high cos2 are in red. Note that the variable categories Co, Ss, Pv and Pb are not very well represented by the first two dimensions. Therefore, the position of the corresponding triangles on the scatter plot should be interpreted with some caution, and an upper dimensional solution is probably necessary. In the mosaic plot of legislative compliance, pipe material and type of water data, the area of each rectangle is proportional to the number of cases in that cell. A mosaic plot was used to visualize data from these qualitative variables to show their proportions or associations (Figure 2b). Additionally, the chi-square test of independence between pipe material and legislative compliance showed a Pearson’s λ value = 9.42 (*p* value = 0.15), so they were not statistically significantly associated (*p* < 0.05), and the null hypothesis was accepted. Figure 2b shows a matrix of pairwise mosaic plots using a variable against every other variable (excepting equipment).

**Figure 3:**
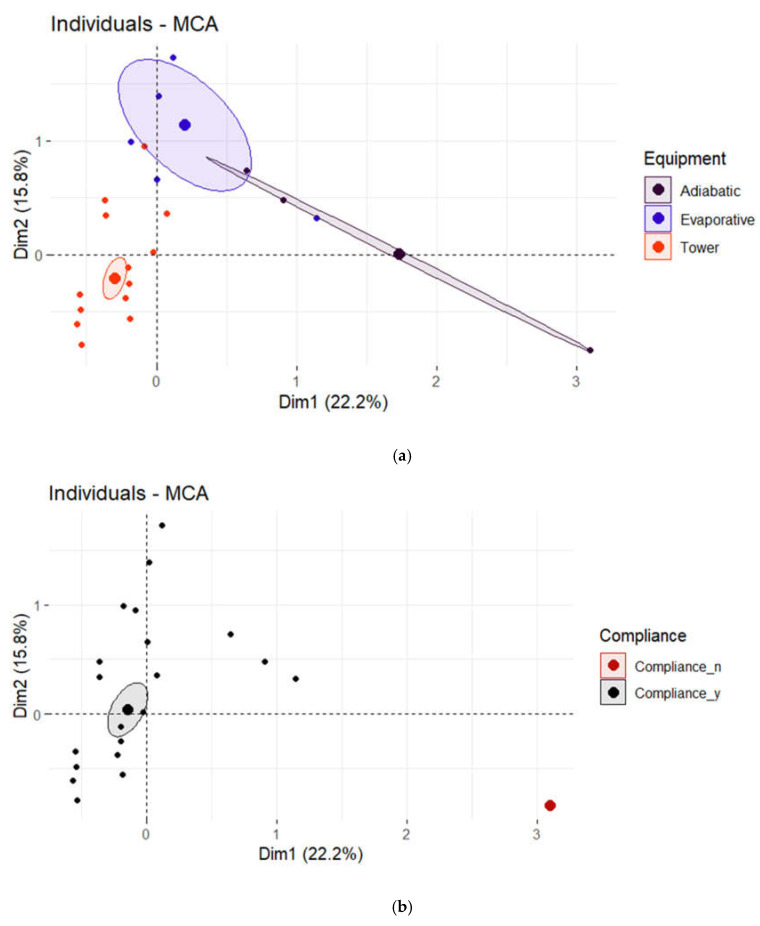

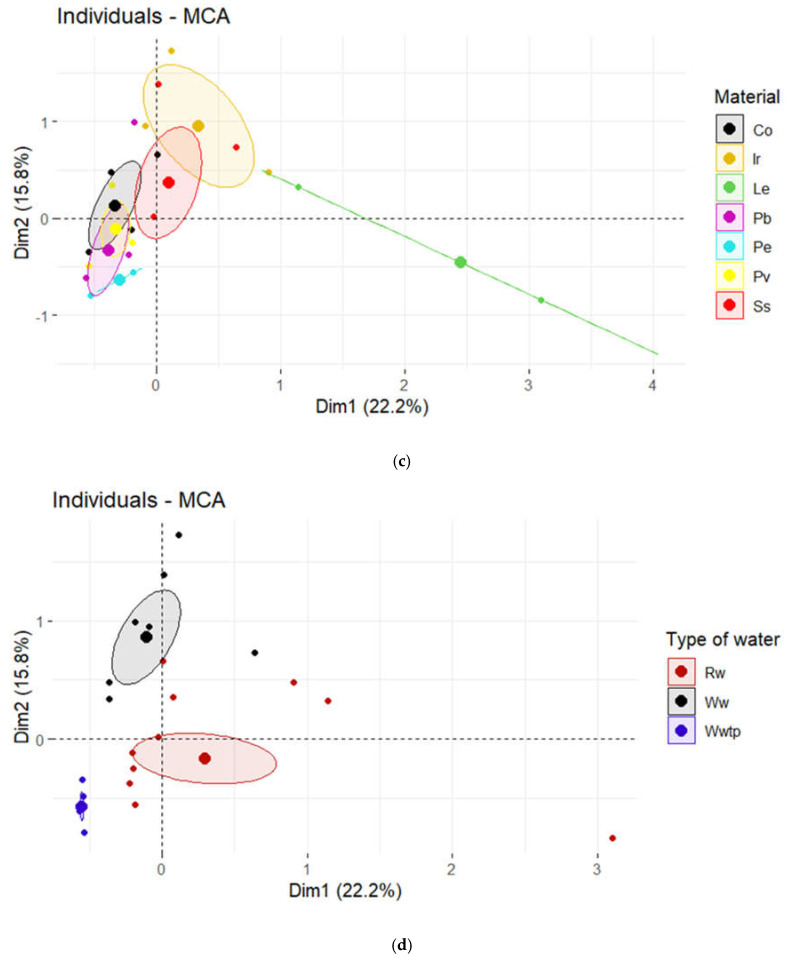
Confidence ellipses around the mean points of categories (Poisson) for: (**a**) equipment; (**b**) legislative compliance; (**c**) pipe material; and (**d**) type of water.

Figure 3 shows graphs of cases for “equipment, legislative compliance, pipe material and type of water”. In each representation, groups of cases were identified according to the different categories of each variable. It is possible to clearly recognize the confidence ellipse clustering cases of each category of a single variable.

## 4. Discussion

Prevention and control of *Legionella* in risk facilities worldwide have been discussed in depth, and new facilities or locations at risk for the development of the bacteria have even been proposed [19]. For these reasons, multiple international regulations have been implemented to maintain standards that allow the appearance and dispersion of biological agents to be controlled [21]. Spain has mandatory regulations for the hygienic/preventive maintenance of risk facilities, although these regulations do not potentially cover all the sources where the bacteria could have a niche. However, legislation has defined a health framework that aims to impact the epidemiology of legionellosis, and although there are many factors that influence the frequency and distribution of the disease, a great deal remains unclear in relation to environmental health [22].

In recent years, several studies have reported associations of pipe materials of installations at risk with colonization and amplification of the bacteria [23,24,25], particularly referring to air–water cooling systems but without identifying the possible role of the type of water transferred. Other studies have referred to the selected area of this study or different devices [26]. Regarding Spanish legislative compliance for the prevention of legionellosis, this research found no relationship between pipe material and type of water. Interestingly, the materials that were most incompatible with the development of bacteria were those that contributed the most to the Pearson’s chi-square statistic and expressed better preventive compliance. This outcome denotes that facilities with Co, Le and Pv should be reviewed regarding maintenance [27,28], especially facilities with Le, since Le is one of the substrates in which the bacteria presents the greatest risk of proliferation [29], although it contributed little to the model. Additionally, Le is a material that is normally associated with old installations and linked to obsolete buildings, so it needs greater attention. Figure 1 shows an association between Ww–Ir and Wwtp–Pb and a strong, positive association between Ww and Ir. Negative residuals indicated that Wwtp and Ww were negatively associated with Co.

The MCA showed that legislative compliance was most correlated with dimension 1 and the type of water with dimension 2. The variable categories of MCA displayed those with a similar profile grouped together. Second, negatively correlated variable categories were positioned on opposite sides of the plot origin (opposing quadrants). Furthermore, category points that were away from the origin were well represented on the factor map (Compliance_n, Le, Adiabatic, Evaporative, Ir, Ww). The cos2 of variables to dimensions 1–2 determined that variable categories, except Co, Ss, Pv, Pb, Pe and Rw, were > 0.4 by the first two dimensions. Compliance_n was primarily related to Le, Adiabatic and Rw and Compliance_y to Co, Pv, Tower and Wwtp.

Confidence ellipses around categories of categorical variables indicated significant differences among categories. In particular, the categories of “equipment” and “type of water” were significantly different from each other. Regarding the “legislative compliance” variable, the categories were significantly different, and regarding the “pipe material” variable, Ir and Ss were significantly different from the others, whereas the other categories were not significantly different from each other.

*Legionella* infection originating in air–water cooling units is generally acquired in community and hospital settings, making it necessary to distinguish between these cases in epidemiological surveillance. Legionellosis can occur in the form of outbreaks and isolated or sporadic cases. In the abovementioned settings, the disease may be associated with various types of facilities, equipment, and buildings. The facilities that are most often contaminated with *Legionella* and have been identified as sources of infection include hot and cold water distribution and evaporative cooling systems (such as cooling towers and evaporative condensers in health centres, hotels or other buildings) [30]. The common denominator is that these units use water at temperatures that allow the proliferation of bacteria and produce aerosols during operation. Variations in water temperature throughout the hydraulic circuit of the installation, together with stagnation and the presence of biofilms or biolayers, calcareous incrustations, corrosion or mineral precipitates, are factors that favour the proliferation of *Legionella* [31,32]. Likewise, the materials that make up the hydraulic circuit must resist the aggressive action of water and chlorine or other disinfectants to avoid corrosion phenomena [33]. Materials that favour the development of bacteria and fungi, such as leather, wood, fibre cement, concrete or cellulose derivatives, should be avoided. These factors can affect the degradation of the pipe substrate and create favourable conditions for the growth of bacteria. Therefore, the state of conservation and general cleanliness must be reviewed to detect the presence of sediments, incrustations, corrosion products, sludge and any other circumstances that alter or may alter the proper functioning of the installation [34].

Additionally, the material of the pipes must be considered a risk factor, as well as other factors that may influence their stability and may increase the risk of bacterial growth. The physicochemical and microbiological conditions of the type of transferred water can affect the presence and development of the bacteria, which together with the type of material and other indicated variables could favour optimal niches for microbial growth. The analysis of the water quality throughout the hydraulic circuit of the installation plays a crucial role in hygienic/preventive maintenance work to control *Legionella*. In fact, water quality analysis is one of the components of the verification process of the effectiveness of the maintenance and revision program and of the water treatment and cleaning and disinfection programs of the installation, as both the sampling points and the moment when the sample is taken are important aspects [35]. As described, water quality is assessed based on microbiological parameters (aerobic and *Legionella* spp.) and physical/chemical parameters that should preferably be analysed in situ (pH, conductivity, temperature, etc.) at the time of sample collection and others that, depending on their analytical complexity or their importance in the adoption of corrective measures, must be determined in the laboratory [36]. Regarding the use of water that does not come from a public or private distribution network, it is estimated that an administrative concession for the use of the resource will be needed, issued by the competent authority in the field of management of the public hydraulic domain.

Concerning the epidemiological control of the bacteria, it is mandatory that the owners and installation companies of air–water cooling units notify the corresponding health administration [37] about their start-ups, the numbers and technical characteristics of the units, and the modifications that affect the systems. Likewise, the licensees must also notify authorities about the definitive cessation of the installation activity. Thus, if the result of the sanitary inspections concludes that there is a risk to public health, the competent sanitary authority may determine the temporary or permanent closure of the facility.

In addition, if necessary, measures can be issued to prevent or minimize the detected risk, including the application of measures related to the specific requirements of the facilities or equipment and the quality of the water [38], the actions of the owner of the installation, and the *Legionella* Prevention and Control Plan. Correction of structural defects, malfunctions or faulty maintenance of the facilities should also be considered.

Finally, the study of the frequency and distribution of disease is presented as a basic tool for reducing the incidence. Due to the ubiquity of the bacterium and its global presence, epidemiological surveillance must be employed according to specific case definition criteria [39]. In addition, a better understanding of the clinical features helps disease control, together with the discovery of the underlying molecular mechanisms of the bacteria, resulting in reductions in morbidity and mortality [40]. The employment of preventive maintenance based on the sources of origin, together with exhaustive control, will comply with containment strategies for the agent and, under certain conditions, result in elimination of *Legionella*.

Legionnaires’ disease is likely to be underrecognized in many countries. Due to the widespread existence of *Legionella pneumophila* in natural and artificial water environments and the lack of cross-protection against different strains, this bacterium is a potentially serious threat to human health. Therefore, effective monitoring of the virulence of this biological agent in the water environment is very important to prevent and control the disease [41]. Understanding the virulence of *Legionella pneumophila* not only can help to predict the risk of possible outbreaks in advance but also can enable more targeted clinical treatment. Comprehending the epidemiology and ecology of *Legionella pneumophila* isolated from public facilities in terms of public health and biology is a very important duty [42,43]. Due to the potential for water sources to harbour and disseminate *Legionella pneumophila* and to the influence of geographical conditions on the virulence of the bacterium, timely and accurate virulence surveillance is urgently needed [44].

This study showed that comprehensive compliance with Spanish health legislation was not observed, so there was potential development of *Legionella pneumophila*, and significant differences related to the prevention and control of legionellosis may be linked to pipe materials and types of water [45]. The aforementioned regulations should include specific references to these variables, which are compatible with the maintenance of risk facilities and physical/chemical and microbiological water control [46,47]. Additionally, it is mandatory to characterize air–water cooling units and their associations with easily colonized pipe materials, water that has not been treated and devices not in legislation compliance [48]. To reduce the risk of legionellosis, Spanish health legislation must be adhered to, and sanitary inspections must be increased. Finally, more studies related to the present one should be performed in different geographical areas and distinct risk facilities with more environmental variables and diverse climatic conditions.

## Data Availability

All of the data generated or analysed during this study are included in this published article and are available upon reasonable request to the corresponding author.

## Limitations

This research is subject to potential information biases. Further studies are necessary to better understand the associations among pipe material, type of water and legionellosis. The microbiological and chemical qualities of both well and recycled water vary greatly, and well water can originate from different groundwater sources. The age of pipes is usually a factor that can cause contamination and biofilms that may protect *Legionella,* but in this study, this factor was unknown. The implementation of sanitary criteria for the prevention and control of legionellosis could be different in other provinces or autonomous communities of Spain. Environmental surveillance for the prevention of legionellosis may not cover all risk units due to lack of communication from the owners with the corresponding authority.

## References

[1] World Health Organization (WHO) Legionellosis-Argentina. https://www.who.int/emergencies/disease-outbreak-news/item/2022-DON407.

[2] Centers for Disease Control and Prevention (CDC) Legionella (Legionnaires’ Disease and Pontiac Fever). https://www.cdc.gov/legionella/about/history.html.

[3] Barimani M.J. (2022). Legionella: An uncommon cause of community-acquired pneumonia. JAAPA.

[4] Principe L., Tomao P., Visca P. (2017). Legionellosis in the occupational setting. Environ. Res..

[5] Cunha B.A., Burillo A., Bouza E. (2016). Legionnaires’ disease. Lancet.

[6] Herwaldt L.A., Marra A.R. (2018). Legionella: A reemerging pathogen. Curr. Opin. Infect. Dis..

[7] Kanarek P., Bogiel T., Breza-Boruta B. (2022). Legionellosis risk-an overview of Legionella spp. habitats in Europe. Environ. Sci. Pollut. Res. Int..

[8] Hamilton K.A., Hamilton M.T., Johnson W., Jjemba P., Bukhari Z., LeChevallier M., Haas C.N. (2018). Health risks from exposure to Legionella in reclaimed water aerosols: Toilet flushing, spray irrigation, and cooling towers. Water Res..

[9] Paschke A., Schaible U.E., Hein W. (2019). Legionella transmission through cooling towers: Towards better control and research of a neglected pathogen. Lancet Respir. Med..

[10] Jalili M., Ehrampoush M.H., Zandi H., Ebrahimi A.A., Mokhtari M., Samaei M.R., Abbasi F. (2021). Risk assessment and disease burden of Legionella presence in cooling towers of Iran’s central hospitals. Environ. Sci. Pollut. Res. Int..

[11] Walser S.M., Gerstner D.G., Brenner B., Höller C., Liebl B., Herr C.E. (2014). Assessing the environmental health relevance of cooling towers-a systematic review of legionellosis outbreaks. Int. J. Hyg. Environ. Heal..

[12] Royal Decree 487/2022, of June 21, which establishes the health requirements for the prevention and control of legionellosis. B.O.E. num. 148 of June 22, 2022.

[13] Ariyadasa S., Daear W., Abeysekera G., Billington C., Fee C., Prenner E., Pang L. (2022). Evaluation of biopolymer materials and synthesis techniques to develop a rod-shaped biopolymer surrogate for Legionella pneumophila. Polymers.

[14] Cullom A.C., Martin R.L., Song Y., Williams K., Williams A., Pruden A., Edwards M.A. (2020). Critical review: Propensity of premise plumbing pipe materials to enhance or diminish growth of Legionella and other opportunistic pathogens. Pathogens.

[15] Rakić A., Vukić Lušić D., Jurčev Savičević A. (2022). Influence of metal concentration and plumbing materials on Legionella contamination. Microorganisms.

[16] (2017). Water quality-Enumeration of Legionella.

[17] (1999). Water Quality-Enumeration of Culturable Micro-Organisms-Colony Count by Inoculation in a Nutrient Agar culture Medium.

[18] Gea-Izquierdo E. (2022). Assessment of Legionella pneumophila development through irrigation system materials. App. Ecol. Environ. Res..

[19] Schwake D.O., Alum A., Abbaszadegan M. (2021). Legionella occurrence beyond cooling towers and premise plumbing. Microorganisms.

[20] Kassambara A. (2017). Principal Guide to Principal Component Methods in R: Practical Guide.

[21] Van Kenhove E., Dinne K., Janssens A., Laverge J. (2019). Overview and comparison of Legionella regulations worldwide. Am. J. Infect. Control..

[22] Serrano-Suárez A., Dellundé J., Salvadó H., Cervero-Aragó S., Méndez J., Canals O., Blanco S., Arcas A., Araujo R. (2013). Microbial and physicochemical parameters associated with Legionella contamination in hot water recirculation systems. Environ. Sci. Pollut. Res. Int..

[23] Learbuch K.L.G., Smidt H., van der Wielen P.W.J.J. (2021). Influence of pipe materials on the microbial community in unchlorinated drinking water and biofilm. Water Res..

[24] Lu J., Buse H.Y., Gomez-Alvarez V., Struewing I., Santo Domingo J., Ashbolt N.J. (2014). Impact of drinking water conditions and copper materials on downstream biofilm microbial communities and Legionella pneumophila colonization. J. Appl. Microbiol..

[25] Rhoads W.J., Pruden A., Edwards M.A. (2017). Interactive effects of corrosion, copper, and chloramines on Legionella and Mycobacteria in hot water plumbing. Environ. Sci. Technol..

[26] Gea-Izquierdo E. (2020). Biological risk of Legionella pneumophila in irrigation systems. Rev. Salud Publica (Bogota).

[27] Bédard E., Trigui H., Liang J., Doberva M., Paranjape K., Lalancette C., Allegra S., Faucher S.P., Prévost M. (2021). Local adaptation of Legionella pneumophila within a hospital hot water system increases tolerance to copper. Appl. Environ. Microbiol..

[28] Buse H.Y., Morris B.J., Struewing I.T., Szabo J.G. (2019). Chlorine and Monochloramine Disinfection of Legionella pneumophila Colonizing Copper and Polyvinyl Chloride Drinking Water Biofilms. Appl. Environ. Microbiol..

[29] Molino P.J., Bentham R., Higgins M.J., Hinds J., Whiley H. (2019). Public Health Risks Associated with Heavy Metal and Microbial Contamination of Drinking Water in Australia. Int. J. Environ. Res. Public. Heal..

[30] Passot F., Peslier S., Benzinger M.J., Blackburn J., Thompson W., Bastin B., Dumont A., Dukan S. (2022). Validation of MICA Legionella for Enumeration of Legionella pneumophila in sanitary waters and cooling tower waters: AOAC Performance Tested MethodSM #032201. J. AOAC Int..

[31] Martin R.L., Strom O.R., Pruden A., Edwards M.A. (2020). Interactive Effects of Copper Pipe, Stagnation, Corrosion Control, and Disinfectant Residual Influenced Reduction of Legionella pneumophila during Simulations of the Flint Water Crisis. Pathogens.

[32] Marchesi I., Ferranti G., Mansi A., Marcelloni A.M., Proietto A.R., Saini N., Borella P., Bargellini A. (2016). Control of Legionella Contamination and Risk of Corrosion in Hospital Water Networks following Various Disinfection Procedures. Appl. Environ. Microbiol..

[33] Martin R.L., Harrison K., Proctor C.R., Martin A., Williams K., Pruden A., Edwards M.A. (2020). Chlorine Disinfection of Legionella spp., L. pneumophila, and Acanthamoeba under Warm Water Premise Plumbing Conditions. Microorganisms.

[34] Lu J., Struewing I., Yelton S., Ashbolt N. (2015). Molecular survey of occurrence and quantity of Legionella spp., Mycobacterium spp., Pseudomonas aeruginosa and amoeba hosts in municipal drinking water storage tank sediments. J. Appl. Microbiol..

[35] Ali S., Phillips C.A., Phillips P.S., Bates M. (2010). Isolation and identification of Legionella pneumophila from material reclamation facilities. Int. J. Environ. Heal. Res..

[36] Campaña M., Del Hoyo R., Monleón-Getino A., Checa J. (2023). Predicting Legionella contamination in cooling towers and evaporative condensers from microbiological and physicochemical parameters. Int. J. Hyg. Environ. Heal..

[37] Hauswirth S. (2011). Revision of the drinking water regulations. Gesundheitswesen.

[38] Lasheras A., Boulestreau H., Rogues A.M., Ohayon-Courtes C., Labadie J.C., Gachie J.P. (2006). Influence of amoebae and physical and chemical characteristics of water on presence and proliferation of Legionella species in hospital water systems. Am. J. Infect. Control..

[39] Barskey A.E., Derado G., Edens C. (2022). Rising Incidence of Legionnaires’ Disease and Associated Epidemiologic Patterns, United States, 1992–2018. Emerg. Infect. Dis..

[40] Gerace E., Mancuso G., Midiri A., Poidomani S., Zummo S., Biondo C. (2022). Recent Advances in the Use of Molecular Methods for the Diagnosis of Bacterial Infections. Pathogens.

[41] Ji P., Parks J., Edwards M.A., Pruden A. (2015). Impact of Water Chemistry, Pipe Material and Stagnation on the Building Plumbing Microbiome. PLoS ONE.

[42] Falkinham J.O., Hilborn E.D., Arduino M.J., Pruden A., Edwards M.A. (2015). Epidemiology and Ecology of Opportunistic Premise Plumbing Pathogens: Legionella pneumophila, Mycobacterium avium, and Pseudomonas aeruginosa. Environ. Heal. Perspect..

[43] Phin N., Parry-Ford F., Harrison T., Stagg H.R., Zhang N., Kumar K., Lortholary O., Zumla A., Abubakar I. (2014). Epidemiology and clinical management of legionnaires’ disease. Lancet Infect. Dis..

[44] Qin T., Zhao D., Zhu L., Ren H., Li Y., Liu X., Li X., Li W., Zhao N., Lu J. (2022). Legionella pneumophila risk from cooling tower systems in China. Appl. Environ. Microbiol..

[45] Sawczyn-Domańska A. (2021). Detection of Legionella spp. and occurrence of virulence genes: lvh, rtxA and enhC in water samples from artificial water systems. Ann. Agric. Environ. Med..

[46] Grace R.D., Dewar N.E., Barnes W.G., Hodges G.R. (1981). Susceptibility of Legionella pneumophila to three cooling tower microbicides. Appl. Environ. Microbiol..

[47] Macleod-Smith R.I. (2003). Controlling Legionella without damaging evaporative condensers. Heal. Estate.

[48] Crook B., Willerton L., Smith D., Wilson L., Poran V., Helps J., McDermott P. (2020). Legionella risk in evaporative cooling systems and underlying causes of associated breaches in health and safety compliance. Int. J. Hyg. Environ. Heal..

